# Plasma-activated water modulates taxanes production and phenylalanineammonia-lyase activity in *Taxus baccata* cell culture

**DOI:** 10.1371/journal.pone.0325518

**Published:** 2025-07-29

**Authors:** Mojan Haji Fathali Tehrani, Mohtaram Mahmoudieh, Meisam Zargar, Mohammad Hossein Mirjalili, Mahsa Bamneshin, Mohammad Reza Naghavi

**Affiliations:** 1 Division of Biotechnology, Department of Agronomy and Plant Breeding, College of Agricultural and Natural Resources, University of Tehran, Karaj, Iran; 2 Department of Agrobiotechnology, Agrarian Technological Institute, RUDN University, Moscow, Russia; 3 Department of Agriculture, Medicinal Plants and Drugs Research Institute, Shahid Beheshti University, Tehran, Iran; Istanbul Arel University: Istanbul Arel Universitesi, TÜRKIYE

## Abstract

Paclitaxel, an anti-cancer compound from the *Taxus baccata* L. (yew tree), is limited in availability from natural sources. This study explores the use of plasma-activated water (PAW) as an elicitor in *T*. *baccata* suspension cell cultures to enhance the production of paclitaxel and its precursor, 10-Deacetylbaccatin III (10-DAB III). The effects of PAW on various factors, including fresh and dry weight, cell viability, and phenylalanine ammonia-lyase (PAL) activity were investigated. The PAW treatment was conducted at different concentrations (200, 300, and 400 μL), with a pH of 5.6 to 5.8, and was applied at different time points (0, 7, 14, and 21 days). The results revealed that 10-DAB III was increased (14.04 µg/g) significantly at a concentration of 400 μL of PAW on day 21. In contrast, the highest paclitaxel content (3.342 µg/g) was achieved in the control group on day 21. The PAW treatment reduced cell viability by 32.25% compared to day 0 (86.25%), and PAL activity increased initially before declining, but remained higher than in the control group. This study is the first to demonstrate the potential of PAW to enhance taxanes production in *T*. *baccata* cell cultures, warranting further investigation into the underlying mechanisms.

## Introduction

**Fig 1:**
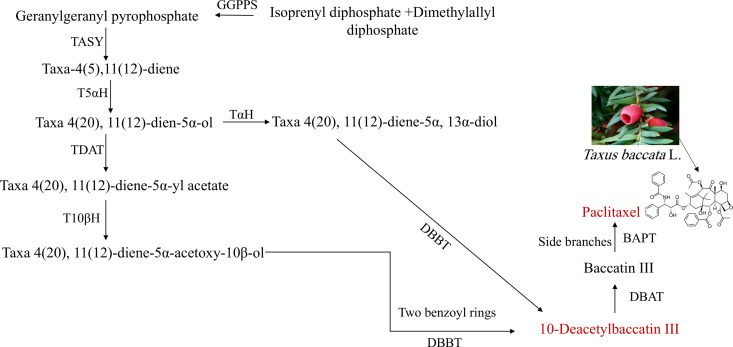
Schematic diagram of the main steps of the paclitaxel biosynthesis pathway. The abbreviations are as follows: GGPPS, Geranyl pyrophosphate synthase; TASY, taxadiene synthase; T5αOH, taxadiene 5α hydroxylase; TDAT, taxadiene 5α-ol O-acetyltransferase; T10βOH, Taxol 10β hydroxylase; TαH, taxadiene 13a-hydroxylase; DBBT, taxane 2a-O-benzoyl-transferase; DBAT, 10-deacetylbaccatin III-10-O-acetyltransferase; BAPT, baccatin III: 3-amino, 3-phenylpropanoyltransferase.

*Taxus baccata* L. (yew tree) belongs to the Taxaceae family and has a chromosome number of 2n = 2x = 24. This species is a long-lived, evergreen tree native to Europe and include eight different varieties [1]. *T*. *baccata* primarily inhabits the eastern regions of Mazandaran and Golestan provinces in Iran [2]. Paclitaxel is a tricyclic diterpenoid anti-cancer compound first discovered and extracted from the bark of *T*. *brevifolia* Nutt. in 1966 [3–5]. This anti-cancer agent is used for the treatment of various types of cancer, such as breast, ovarian and bone cancers [6]. The biosynthesis of paclitaxel occurs in three distinct stages (early, middle, and late) and involves complex oxidation and acylation reactions. Initially, geranylgeranyl pyrophosphate (GGPP) and phenylalanine are synthesized from isoprenyl diphosphate (IPP) and dimethylallyl diphosphate (DMAPP) via the activity of geranylgeranyl pyrophosphate synthase (GGPPS) [7,8]. GGPP which is a crucial precursor in the paclitaxel biosynthesis pathway, is converted into taxadiene or taxa 4(5), 11(12)-dien with the aid of the taxadiene synthase (TASY) enzyme [7,9]. Another isomer of taxadine called taxa-4(20),11(12) dine is created hydroxylated and converted into the alcohol compound taxadinol or taxa 4(20), 11(12)-Dien-5α-ol by taxadiene 5α-hydroxylase (T5αH). In one reaction, taxadiene 13α-hydroxylase (TαH) converts taxa 4(20), 11(12)-dien-5α-ol into taxa 4(20), 11(12)-diene-5α, 13α-diol which can then be converted to 10-deacetyl baccatin III with the help of the enzyme taxane 2α-O-benzoyltransferase [10,11]. In an alternative reaction, taxadiene 5α-ol O-acetyltransferase (TDAT) transforms taxa 4(20), 11(12)-diene-5α-ol into taxa 4(20), 11(12)-diene-5α-yl acetate. This compound is then hydroxylated at C10 by taxane 10β-hydroxylase (T10βH), yielding taxa 4(20), 11(12)-diene-5α-acetoxy-10β-ol [10,12]. The formation of 10-Deacetyl baccatin III (10-DAB III) is occurred with the assistance of taxane 2α-O-benzoyltransferase (DBBT) [3].Subsequently, 10-acetylbaccatin III is converted to baccatin III by 10-acetylbaccatin III acetyltransferase (DBAT) followed by production of paclitaxel by the enzyme baccatin III, 3-anino, 3-phenylpropanoyltransferase (BAPT) [6,7] (Fig 1).

Although, the process of extracting paclitaxel is lengthy, the yield is minimal (0.01% of dry weight) [13–15]. Among the various methods for producing paclitaxel (isolation from wild or plantation trees, total chemical synthesis, semi-synthesis, and the use of yew endophytic fungi) the most promising approach is the industrial cultivation of *Taxus* species cell cultures. Acquiring this compound or a semisynthetic precursor from *Taxus* trees is not environmentally sustainable due to their limited natural abundance, and chemical synthesis is not economically viable. Therefore, the preferred method is the biotechnological production of paclitaxel using optimized cell cultures from *Taxus* species, as these cell suspensions can be grown under controlled and cost-effective conditions. [16,17].

Plant cell culture has emerged as an effective industrial and commercial approach for the production of paclitaxel [18,19]. As reviewed by Yin et al., [20], the cell suspension culture offers several advantages for biosynthetic methods, particularly in the production of paclitaxel. It allows the entire process to occur in a bioreactor, enabling large-scale, simultaneous production that is environmentally friendly. The cells of *T*. *chinensis* Pilg. have a complete paclitaxel metabolism pathway and proliferate in the reactor, therefore, the entire platform is sustainable. Additionally, high-yield cell lines created through genetic manipulation and the use of multiple inducers can significantly boost production. This method represents a promising new direction for industrial paclitaxel production, following the semisynthetic extraction from *Taxus* plants [20]. The concept was first introduced in a patent application, achieving initial yields of 1–3 mg/L [21]. Since then, the maximum yield has reached 565 mg/L through several key approaches: modifying the culture medium and carbon source, fine-tuning culture conditions, using inducers to enhance secondary metabolite production, and applying genetic modifications [20]. However, this method does not necessarily guarantee an increase in the production rate [22,23].

To tackle this challenge, the use of elicitors has proven to be an effective strategy for boosting production of specific plant metabolites in cell suspension cultures [24]. Elicitors are categorized into biotic and abiotic sources. Abiotic elicitors, which derive from non-biological origins, include mineral compounds (such as heavy metal salts, metal ions, and metal oxides) and physical stressors (like temperature changes, cold shock, UV radiation, osmosis, water/light stress, and salinity) [25]. Biological elicitors, on the other hand, originate from living sources and can be either exogenous or endogenous. Exogenous elicitors, such as chitosan, plant polysaccharides, and oligosaccharides, trigger plant defense responses. Endogenous elicitors, such as stress-induced salicylic acid, are recognized by cell membrane receptors, promoting phytoalexin production. Both types enhance secondary metabolite production in plant cell cultures, reducing processing time and increasing yields of secondary metabolites [26].

Different elicitors were investigated on the production of valuable secondary metabolites, particularly taxanes [20]. The effects of light and copper sulfate as elicitors on the biosynthesis of certain taxanes in *T. baccata* cell cultures were investigated. The results indicated that light increased the level of both paclitaxel and 10-DAB III while, copper sulfate reduced the level of paclitaxel, but increased 10-DAB III [13,27]. Recently, our study revealed that the elicitors derived from *Fusarium graminearum* enhanced the production of 10-DAB III and paclitaxel in *T*. *baccata* suspension cell cultures [28]. The effectiveness of elicitors is grounded in the idea that plant cells under stress situations, carbon is redirected from producing biomass to synthesizing secondary compounds as a means of defense [26,29,30].

Recently, researchers explored an innovative approach using plasma activated water (PAW) to simultaneously boost food security and minimize the environmental impact of agriculture [31]. Plasma-activated water (PAW) is rich in long-lived reactive oxygen and nitrogen species (RONS), such as hydrogen peroxide and nitrates, which play key roles in signaling pathways that regulate plant metabolism, development, and stress responses [32]. Exposing water to cold atmospheric pressure plasma generates reactive oxygen and nitrogen species in the liquid phase [32]. In recent years, it has been proven that PAW treatment enhances plant growth, and several pioneering experiments have focused on the mechanisms triggered by this treatment in inducing resistance in plants [31,33,34].

In response to both abiotic and biotic stresses, plants activate an adaptive defense mechanism that generates RONS. These RONS serve as crucial signaling molecules, triggering the expression of defense-related genes [35]. Furthermore, the bursts of RONS induced by these stresses stimulate the synthesis of secondary metabolites, which often act as precursors for defense hormones within plant cells [31]. A review study reported that elicitor treatment may have triggered the production of ROS as a response to stress leading to enhance their antioxidant enzyme activity and production of secondary metabolites [36]. William Crookes first identified plasma in 1879, and it makes up 99% of the visible universe [37]. There are two types of plasma: thermal plasma and non-thermal or cold atmospheric plasma. In plasma, the thermal movement of ions and magnetic forces can be disregarded, meaning only electric force influence the particles, with no pressure force involved [37]. The exposure of water to cold atmospheric pressure plasma leads to the generation of RONS in the liquid phase [31–34,38].

Zambon et al., [31] conducted a study on micropropagated periwinkle shoots and grapevine plants treated with PAW. They analyzed the differential expression of genes associated with key plant defense pathways. Their findings indicated that PAW effectively stimulates plant defense responses [31]. Kim et al., [39] reported that the treatment radish sprouts with PAW led to an increase in the levels of indolic and aliphatic glucosinolates, as well as a significant increase in phenolic content specifically, a 288.8% increase compared to the control after 30 min of plasma treatment. Indeed, their finding showed that PAW had a positive effect on the production of these secondary metabolites, suggesting that it enhances the functional components of plants during hydroponic cultivation. While PAW improves germination and increases certain metabolites, it negatively affects plant growth by oxidizing the roots. This dual effect indicates that, although PAW can enhance secondary metabolite levels, it may also lead to growth disorders, warranting further research to fully understand its implications [39]. In cell suspension cultures, researchers can potentially harness the benefits of PAW while mitigating its adverse effects on plant growth. However, a significant challenge lies in identifying an optimized treatment duration and PAW concentration, to ensure cell viability while enhancing the production of secondary metabolites [39].

To counteract the effects of RONS produced upon PAW treatment, plants have evolved antioxidant defense mechanisms, including PAL, which play a crucial role in enhancing overall antioxidant activity [40]. PAL is an important enzyme in the phenylpropanoid pathway and is frequently upregulated in response to both biotic and abiotic stresses [27]. According to Han and Yuan. [41] the activation of PAL activity and the accumulation of phenolic compounds are regulated by the oxidative burst observed in the suspension culture of *T. cuspidate* Siebold & Zucc. [41]. This process is thought to alter membrane permeability, subsequently inducing secondary metabolism.

In our previous study, the effects of white light and 2-aminoindan-2-phosphonic acid (AIP), a phenylalanine ammonia-lyase (PAL) inhibitor, on *T*. *baccata* cell suspension culture was demonstrated [27]. Light-induced activation of PAL transcription and changes in the expression of relevant biosynthetic genes can reduce cell growth while increasing the content of total phenolic compounds and taxanes. In addition, light significantly enhanced the expression of *PAL*, *DBTNBT*, *BAPT*, and *T13αOH* genes, but did not affect *DXS* expression [27]. The results of our recent study revealed that elicitors derived from *F*. *graminealrum* influenced phenylpropanoid metabolism in a concentration-dependent manner leading to a differential PAL activity among treatments [28].

In this study, the potential of PAW as an innovative elicitor to boost the production of valuable taxanes in plant cell cultures is explored. Indeed, the impact of PAW on the production of paclitaxel, 10-DAB III, as well as on cell growth and PAL activity in treated cell suspension culture of *T. baccata* was evaluated. Utilizing this elicitor represents a distinct approach important in the pharmaceutical industry for production of anti-cancer agents, potentially offering new insights into optimizing paclitaxel production. To best of our knowledge it is the first study that emphasized the significance of potential applications of PAW as an elicitor in the production of taxanes through *T*. *baccata* suspension culture.

## Materials and Methods

### Chemicals and reagents

All general chemicals and reagents used in this study were obtained in analytical grade and purchased at the highest purity available from suppliers (e.g., Sigma Aldrich, Merck and etc.).

### Plant materials and culture condition

Young and fresh branches of the *T. baccata* L. tree was collected from the Botanical Garden of the University of Tehran, Karaj, Iran (35° 48’ N, 50° 57’ E), followed by the formal identification of the plant material by taxonomists at the Department of Horticultural Science, College of Agriculture and Natural Resources, University of Tehran. The surface sterilization of explants (stems) was performed briefly as follows: initially the collected explants were washed with tap water for 1 hour to remove any surface contaminants, followed by a brief immersion in 70% ethanol for 30 seconds. After treating the explants with 0.1% mercury chloride solution for 10 min, they were rinsed twice with autoclaved double-distilled water. Finally, the explants were treated with 2% (v/v) sodium hypochlorite for 15 min, followed by three rinses with sterilized double-distilled water to remove any trace of sodium hypochlorite [28], and used for subsequent experiments.

### Callus induction and suspension cell culture

Sterilized stems (1–1.5 centimeters) were cultured on the Gamborg medium (B5) medium [28,42] supplemented with 4 mg/L 2,4-Dichlorophenoxyacetic acid (2,4-D) and 0.5 mg/L kinetin (pH 5.8), and then kept in the dark for 6 weeks at 25°C. After induction, the calli were transferred to the callus growth and maintenance medium supplemented with 3 mg/L 2,4-D, 0.5 mg/L kinetin, and 0.5 mg/L gibberellic acid (GA_3_), then kept in the dark at 25°C [28].

To prepare and establish a cell suspension culture, 2 g of bright-colored calli were transferred to the 100 mL flasks containing 20 mL media of B5 medium supplemented with 100 mg/L casein, 2 mg/L 1-Naphthaleneacetic acid (NAA), and 0.1 mg/L 6-Benzylaminopurine (BAP), 200 mg/L antioxidant (L-glutamine), 50 mg/L ascorbic acid, and 50 mg/L citric acid. The callus was sub-cultured in liquid medium (pH 5.8) at an agitation speed of 60 rpm in the dark at 25° C to establish a cell suspension culture [28]. Each flask served as an experimental unit, and a total of three flasks were considered as experimental units for each treatment.

### Plasma-activated water preparation and elicitation of cell suspension culture

Plasma-activated water (PAW) was prepared using a GAD plasma machine (Plasma Tech 15B Super Arc System, Iran) with the following specifications: voltage = 220V, frequency = 50 Hz, output power = 2800W, plasma width = 40 mm, plasma length = 110 mm. The machine had dimensions of 65 × 45 × 55 cm and was designed for use with nonconductive materials and PAW [43]. 200 mL of distilled water was poured into a flat container and placed under the glide arc machine at a distance of 2 cm for 6−8 seconds (pH 5.6–5.8). Methyl jasmonate was used as a base elicitor in all B5 production medium including control medium and served as the reference point for all analyses and comparisons in the study. The flasks were placed on a shaker at 60 rpm at 25°C for 21 days [27]. Additionally, a cell growth test was done to determine the best time point for treatment. To assess the PAW impact on the desired product’s performance, the suspension cultures were treated with different concentrations of filtered PAW (200, 300, 400 µL) on the day 7 with three replicates [42].

### Biomass measurements and cell viability

To evaluate the cell growth and viability, the *T*. *baccata* cells were harvested at different time points (0, 7, 14, and 21) after treatment. The culture medium was removed using a Büchner funnel and the fresh weight of the cells was measured. The harvested cells were dried for 48 hours by freeze dryer (LTE Scientific model Lyotrap-Plus™) and the dry weight (DW) of the cells was determined. To assess the cell viability, the samples were incubated for 5 minutes in B5 medium supplemented with 0.01% (w/v) propidium iodide for dead cell labeling and 0.01% (w/v) fluorescein diacetate for live cell labeling. Fluorescence was observed using a fluorescence microscope (Leica DMIRE2, Leica Microsystems Inc., Wetzlar, Germany) connected to a Leica DFC360-FX (Wetzlar, Germany) camera with specific filters [16].

### 10-Deacetylbaccatin III and paclitaxel extraction and HPLC analysis

Freeze-dried cells were added in 20 mL of methanol/water solution (9:1) and agitated for 16 hours at 25°C at 60 rpm. The samples were then sonicated for 10 min, vortexed, and filtered through a Buchner funnel. The cell extracts were washed three times with 15 mL of L-hexane and 30 mL of dichloromethane. Dichloromethane was evaporated at 45°C using a rotary evaporator, and the residues were re-dissolved in acetonitrile, filtered through 0.2 μM filter [44,45] and used for HPLC analysis.

The content of paclitaxel and 10-DAB III were measured using a Waters liquid chromatography system (Waters, USA) comprising a separation module (Separations Module model 2695), a dual absorption detector (model 2487), and an autosampler with a 100 μL injection loop. The analysis utilized a C18 analytical column (Eurospher 100–5 C18, 25 cm × 4.6 mm dimensions) [46], employing an isocratic wash with a mixture of acetonitrile and water at a ratio of 55/45. The flow rate was 1 mL/min at 25°C, with a 20 μL sample injection. Each injection was performed twice (n = 2). Standard solutions for paclitaxel and 10-DAB III were prepared in pure acetonitrile to construct the standard curve, spanning a concentration range of 10–200 µg/g. 10-DAB III and paclitaxel were detected at 230 nm wavelength and verified by comparing retention time and UV absorption spectrum with standards. Chromgate software was used for data processing [47].

### Protein extraction and phenylalanine ammonia-lyase activity measurement

The extraction of the protein was carried out as follows: 2 g of cells was homogenized with 1 g PVP, 50 μL of 1 mM dithiothreitol (DTT), and 5 mL of 0.1 M potassium phosphate buffer (pH 8) through ultrasonication at 4°C. The mixture was then centrifuged (Qiagen Model 4–16KS, Germany) at 2000 rpm for 20 min, and the supernatant was used to assess enzyme activity and measure protein quantity [28]. To determine the PAL activity, 150 μL of protein extract was mixed with 650 μL of 0.1 M potassium phosphate buffer (pH 8) and 200 μL of 0.1 M phenylalanine for 1 h at 36 °C [48]. Cinnamic acid content was measured by its absorbance at 290 nm. Finally, the concentration of protein was measured by quantitative protein assay method known as Bradford’s method [49]. Finally, the enzyme activity was expressed as micromoles of cinnamic acid per milligram of protein per hour.

### Statistical analysis

This study was planned as a completely randomized design with three replications (n = 3). The data analysis was performed using two-way analysis of variance (ANOVA) and Duncan’s multiple-range test at 5% probability level. The R software version 4.3.2. was used to generate test findings.

## Results

### Cell growth and biomass production curve

**Fig 2:**
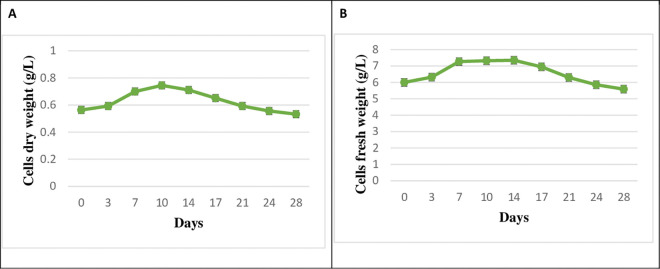
The growth curve and biomass curve of *T. baccata* cells without treatment and under normal conditions. Cells dry weight (g/L) **(A)**, cells fresh weight (g/L) **(B)**. The values are presented as mean ± standard error (SE) of 3 replications (n = 3).

A cell growth test was conducted to determine the optimal day for treatment. The results showed that *T. baccata* cell culture grew during the linear growth phase until day 7 (Fig 2).

### Effect of plasma-activated water on cell growth, biomass production and cell viability of *T. baccata* cell cultures

**Fig 3:**
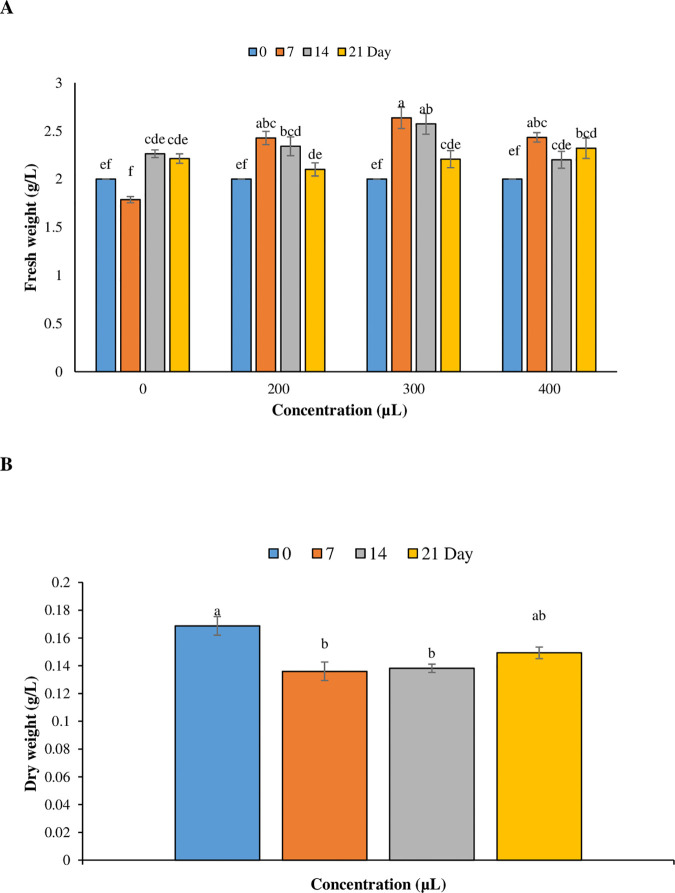
Effects of plasma-activated water on cell growth and biomass of *T*. *baccata* cell cultures. The impact of varying concentrations of plasma-activated water on cell growth **(A)**, and biomass (B) of *T*. *baccata* cell cultures is illustrated. Cell growth and biomass are measured as grams of fresh weight and dry weight per liter respectively. Results are presented as mean ± standard deviation from three replicates (n = 3). Significant differences (*p* < 0.05) are indicated by different letters based on Duncan’s multiple-range tests.

Following elicitation with plasma-activated water (PAW), the impact of different concentrations of PAW (200, 300, 400 μL) was assessed by measuring the fresh weight and dry weight of the cells. The results revealed that PAW enhanced cell growth compared to the control in which the highest fresh weight (2.64 g/L) was achieved at concentration of 300 μL of PAW at day 7, followed by a decline by day 21 (Fig 3A). The effect of concentration, time, and their interaction (concentration × time) of PAW on the fresh weight was significant (*p *< 0.05). In contrast, the effect of PAW concentration and interaction of concentration × time on dry weight were not significant (*p *> 0.05), while the duration of PAW treatment was significant on dry weight. Based on the results, the dry weight at 0, 7, 14, and 21 days after treatment was 0.16875, 0.136, 0.1382, and 0.1493 g/L, respectively (Fig 3B). PAW significantly reduced fresh weight on day 21 at the concentration of 200 μL. However fresh weight showed the highest increase on days 7 and 14 at the concentration of 300 μL PAW. In addition, dry weight was significantly reduced by the day 7, compared to day 0 (Fig 3B).

**Fig 4:**
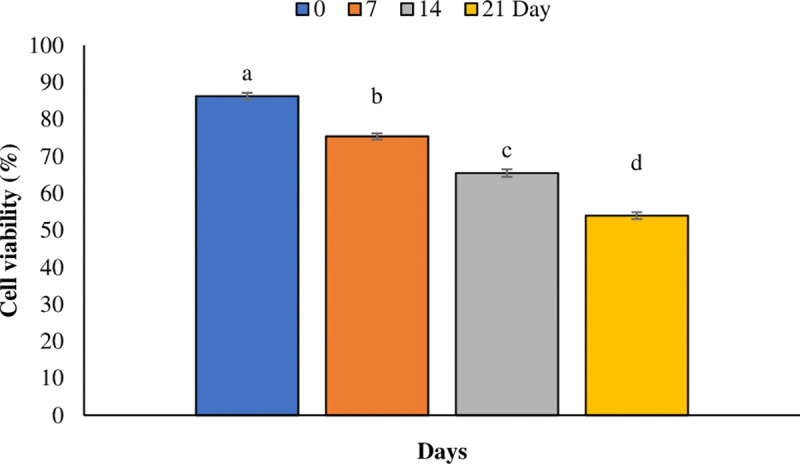
Percentage of cell viability of *T. baccata* treated with plasma-activated water at different time points. The data expressed as the average mean± standard deviation of 3 replications (n = 3). Values that are accompanied by different letters indicate a significant difference (*p* < 0.05) based on Duncan’s multiple-range tests.

The result showed that the cell viability was decreased significantly (*p* < 0.05) over time after PAW treatments. While, concentration had no significant effect on the cell viability percentage. The viability percentages at 0, 7, 14, and 21 days after treatment were 86.25%, 75.375%, 65.5%, and 54%, respectively (Fig 4).

### Effect of plasma-activated water on taxanes production

**Fig 5:**
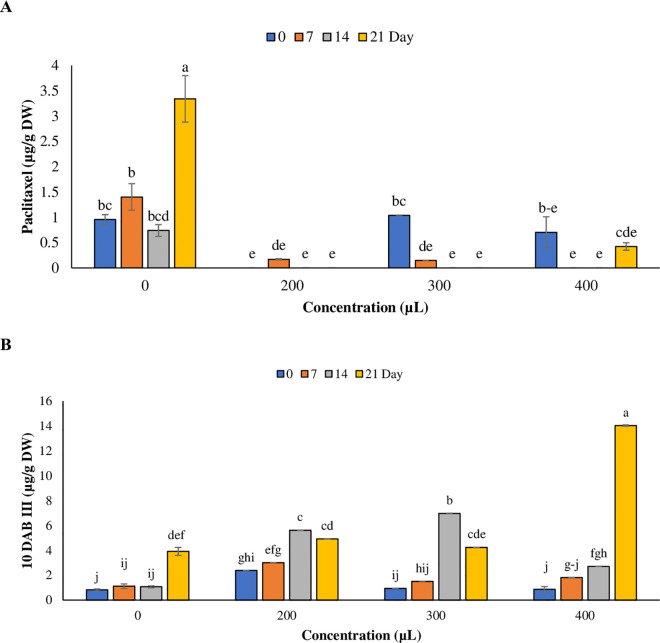
Effects of the plasma-activated water on paclitaxel (A) , and 10-deacetyl baccatin III (10-DAB III) production (µg/g DW) (B) in*T*. *baccata* cell culture in different time points. The values are presented as mean ± standard error (SE). The values followed by different letters are significantly difference (*p* < 0.05) according to Duncan’s multiple-range tests.

Effect of plasma-activated water (PAW) on the levels of paclitaxel and 10-DAB III in *T. baccata* cell cultures was investigated. The results showed that PAW had a significant impact on the amount of paclitaxel and 10-DAB III at the 5% probability level. The highest levels of paclitaxel (3.342 µg/g DW) and 10-DAB III (14.04 µg/g) was occurred on day 21 (1.45-fold, 5.26-fold for paclitaxel and 10-DAB III respectively) in *T. baccata* cell cultures. Notably, the most impactful treatment for enhancing paclitaxel production was observed in the control with no PAW, while 10-DAB III was reached to the highest level at 400 μL PAW. The results indicated that different concentrations of PAW significantly reduced paclitaxel levels compared to the control (Fig 5A), while 10-DAB III levels were increased compared to the control at all time points. (Fig 5B).

### Effect of plasma-activated water on phenylalanine ammonia-lyase activity

**Fig 6:**
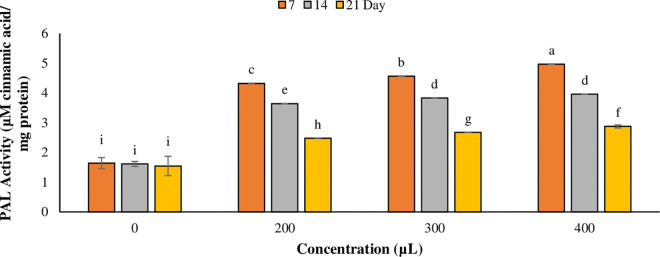
Effect of using plasma-activated water (PAW) on PAL activity in *T. baccata* cells. **Different concentrations of PAW were used as elicitor.** The values are presented as mean ± standard error (SE) of 3 replications (n = 3). The values followed by different letters are significantly difference (*p* < 0.05) according to Duncan’s multiple-range tests.

The results indicated that PAW significantly (*p* < 0.05) enhanced phenylalanine ammonia-lyase (PAL) activity in *T. baccata* cell cultures across all treatments (concentration, time, and their interaction (concentration × time)) on days 7. The highest level of PAL enzyme activity (4.964 cinnamic acid/mg protein) was obtained with 400 μL of PAW at day 7 (Fig 6). Additionally, different concentration of PAW had the positive impact on PAL enzyme activity. Although, by increasing the duration of treatment, PAL activity gradually declined, it was higher in treated cell cultures compared to control.

**Fig 7:**
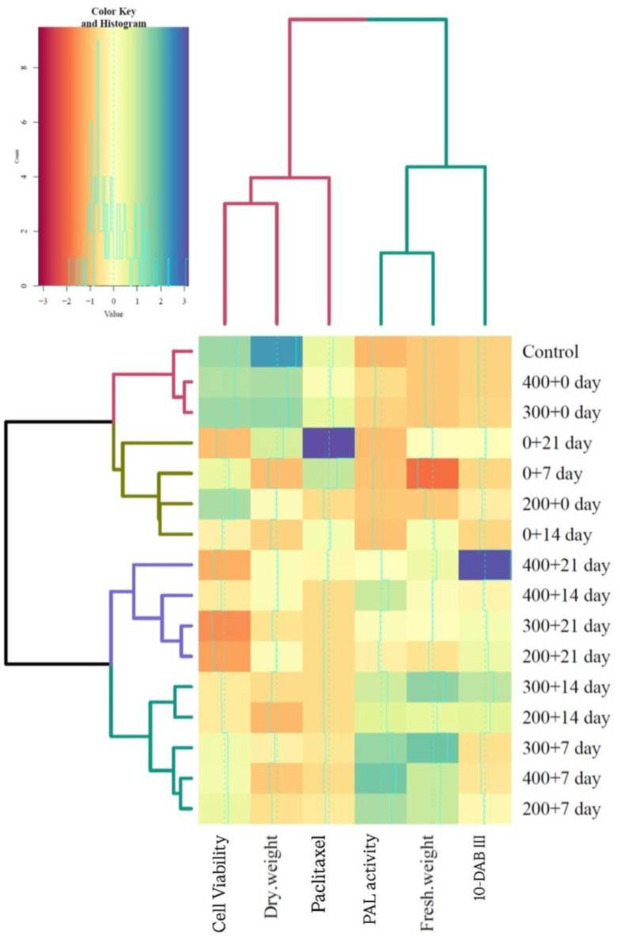
Heatmap of all of the data sets analyzed for plasma-activated water (PAW) treatment. The distribution and intensity of the measured data, including PAL activities, concentrations of paclitaxel and 10-DAB III, cell viability and dry and fresh weight across different concentrations of PAW treatment in different timepoints are presented. The treatments are organized into four distinct groups based on their specific conditions.

A heatmap diagram was created using the Ward method and the squared Euclidean distance to present the distribution and intensity of the measured data across PAW treatments. High-density areas are shown in the dark blue and lower-density areas are indicated in the dark orange (Fig 7).

The treatments are organized into four distinct groups based on their specific conditions. Group I serves as the control and includes the control, 400 + 0 days and 300 + 0 days. Group II consists of treatments that have a duration of 0 days with varying time points (0 + 21 days, 0 + 7 days, 200 + 0 days, 0 + 14 days). Group III includes treatments that have a duration of 21 days, with varying initial values (400 + 21 days, 400 + 14 days, 300 + 21 days, 200 + 21 days). Finally, group IV features’ treatments with a mix of durations, primarily focusing on 7 and 14 days (300 + 14 days, 200 + 14 days, 300 + 7 days, 400 + 7 days, 200 + 7 days). This classification helps visualize the data effectively on the heatmap.

## Discussion

### Elicitor effects on cell growth and biomass of *T. baccata* cell cultures

The rapid cell growth and reliable reproducibility of cells in suspension culture make it an ideal system for large-scale production of valuable secondary metabolites. In this context, developing a suitable cell growth platform is valuable to evaluate the production and accumulation of taxanes [50]. Plasma-activated water (PAW), as an elicitor, is one of the novel strategies for increasing the production of secondary metabolites or their precursor particularly paclitaxel and 10-DAB III which can be used in the semi-synthesis of paclitaxel.

The findings indicate that PAW has a notable impact on cell growth, particularly in terms of fresh weight. The highest fresh weight recorded was 2.64 g/L at a PAW concentration of 300 μL on day 7, which suggests that this concentration is optimal for promoting cell growth during the early stages of treatment. It’s interesting to note that while the fresh weight increased significantly at this concentration, it began to decline by day 21, indicating that the benefits of PAW may be time-sensitive. The statistical analysis shows that both the concentration of PAW and the duration of treatment significantly influenced fresh weight. However, it did not show a significant effect on dry weight, although the duration of treatment alone did have a significant impact. This suggests that while PAW can enhance fresh weight, its effect on dry weight may be more complex and could depend on other factors or longer treatment durations. In different studies, elicitors have been shown to have varying effects on the cell growth and the dry weight. While some studies indicate that elicitors do not negatively impact these parameters [27,51].

The current results are in accordance to previous reports. Some studies reported a reduction in cell biomass and growth upon elicitors application [52,53]. These differences can be attributed to several factors, including the nature of the elicitors used, their concentration, the duration of exposure, plant species, cell lines, and culture conditions involved [51,54,55]. In addition, the reduction in growth was observed in methyl jasmonate-treated *T*. *baccata* cells could be primarily linked to the production of taxanes, rather than a direct effect of methyl jasmonate itself. The variability in taxane concentrations may explain the differences in growth outcomes reported in various studies [56]. Furthermore, taxanes can inhibit cell growth and metabolism when they accumulate in the culture medium and cells due to their own cytotoxicity, leading to reduced production. However, after removing paclitaxel, baccatin III, and other taxanes from the medium, paclitaxel production increased [57]. This emphasizes the importance of considering these strategies for the stagnation caused by accumulations of taxanes in future studies and particularly industrial-scale production.

### Effect of Plasma-activated water on taxanes production in *T. baccata* cell cultures

The changes in taxane production in *T. baccata* cell suspension cultures highlight the significant influence of PAW on their synthesis. However, the production of paclitaxel and 10-DAB III was exhibiting in different manner. For instance, 10-DAB III reached in highest level at 400 μL of PAW on the day 21 in treated cell cultures. While highest amount of paclitaxel production was observed in non-treated cell cultures on the day 21. *T. baccata* cell cultures have shown high 10-DAB III production as a biological response to osmotic stress induced by copper sulfate treatments [13]. These results are in accordance with our finding as the PAW does not positively influence paclitaxel levels; in fact, certain concentrations led to a reduction of this metabolite compared to the control. Although previous research has not investigated the impact of PAW on other taxanes in cell suspension cultures, our finding revealed that applying a concentration of 400 μL PAW on day 21 resulted in a significant increase in 10-DAB III levels. Several studies have indicated a decrease in paclitaxel production due to heightened oxidative degradation in response to severe heat stress, copper sulfate, and fungal elicitors [13,58,59]. It has been shown that despite the increase in paclitaxel precursors, the level of paclitaxel remained unchanged [51,60]. Furthermore, 10-DAB III exhibited a lower degree of toxicity compared to paclitaxel, and consequently, the cell tends to produce this substance [14].

### Effect of plasma-activated water on phenylalanine ammonia-lyase activity

Previous studies have shown that stresses including abiotic stress can cause a reduction in cell viability, while simultaneously increasing phenylalanine ammonia-lyase (PAL) activity [61]. The PAL enzyme is essential for the biosynthesis of secondary compounds such as phenolic acid and flavonoids [62]. In our research, the highest increase in PAL activity in the cell suspension culture of *T. baccata* at a concentration of 400 μL on the day 7 was observed. However, by day 21, PAL activity decreased due to the reduction of cell viability and cell death associated with PAW treatments. In previous study, the fungal elicitors initially induced the activity of PAL in *T. baccata* cell cultures, followed by a subsequent decline in this enzymatic activity [28]. This antioxidant enzyme serves as a defense mechanism, helping to maintain cell viability and promoting survival in stressful conditions [63–65]. The treatment with PAW can trigger a stress response in plant cells, increasing PAL activity, which plays a key role in the plant’s defense by aiding in the production of phenolic compounds that protect against pathogens and environmental stressors [40].

## Conclusion

This study demonstrated that higher concentrations of the plasma-activated water (PAW) increased 10-DAB III levels in *T. baccata* cell cultures. Prolonged treatments of cell cultures to high concentrations of PAW may induce toxicity, and cell death, leading to decreased metabolites production. PAW treatments enhanced fresh weight, but reduced dry weight. PAL activity was increased in response to PAW. Future research needs to investigate a range of PAW concentrations and treatment durations as well as, the synergistic effects of combining PAW with stimulants. The formation of 10-DAB III was associated with apoptosis, contributing to the depletion of paclitaxel. Therefore, it is suggested that future studies should focus on enhancing cell survival to improve paclitaxel production. In addition, further research is essential to explore the effects of PAW treatment on plant defense responses, as well as to identify the specific components of PAW that induce these defense mechanisms. It is worthy to explore the effects of PAW on other secondary metabolites production in other medicinal plants. This understanding is crucial for maximizing taxane concentrations and enhancing their efficacy in therapeutic applications.“
